# *TailTimer*: A device for automating data collection in the rodent tail immersion assay

**DOI:** 10.1371/journal.pone.0256264

**Published:** 2021-08-19

**Authors:** Mallory E. Udell, Jie Ni, Angel Garcia Martinez, Megan K. Mulligan, Eva E. Redei, Hao Chen

**Affiliations:** 1 Department of Pharmacology, Addiction Science, and Toxicology, University of Tennessee Health Science Center, Memphis, TN, United States of America; 2 Department of Genetics, Genomics, and Informatics, University of Tennessee Health Science Center, Memphis, TN, United States of America; 3 Department of Psychiatry and Behavioral Sciences, and Physiology, Northwestern University, Feinberg School of Medicine, Chicago, IL, United States of America; Belgrade University Faculty of Medicine, SERBIA

## Abstract

The tail immersion assay is a widely used method for measuring acute thermal pain in a way which is quantifiable and reproducible. It is non-invasive and measures response to a stimulus that may be encountered by an animal in its natural environment. However, quantification of tail withdrawal latency relies on manual timing of tail flick using a stopwatch, and precise temperatures of the water at the time of measurement are most often not recorded. These two factors greatly reduce the reproducibility of tail immersion assay data and likely contribute to some of the discrepancies present among relevant literature. We designed a device, *TailTimer*, which uses a Raspberry Pi single-board computer, a digital temperature sensor, and two electrical wires, to automatically record tail withdrawal latency and water temperature. We programmed *TailTimer* to continuously display and record water temperature and to only permit the assay to be conducted when the water is within ± 0.25°C of the target temperature. Our software also records the identification of the animals using a radio frequency identification (RFID) system. We further adapted the RFID system to recognize several specific keys as user interface commands, allowing *TailTimer* to be operated via RFID fobs for increased usability. Data recorded using the *TailTimer* device showed a negative linear relationship between tail withdrawal latency and water temperature when tested between 47–50°C. We also observed a previously unreported, yet profound, effect of water mixing speed on latency. In one experiment using *TailTimer*, we observed significantly longer latencies following administration of oral oxycodone versus a distilled water control when measured after 15 mins or 1 h, but not after 4 h. *TailTimer* also detected significant strain differences in baseline latency. These findings valorize *TailTimer* in its sensitivity and reliability for measuring thermal pain thresholds.

## Introduction

Extant research has utilized an assortment of methods to measure and understand nociception relative to the specific type of pain being assessed. Both acute and chronic pain can be modeled in rodents. Moreover, pain can be induced using various types of stimuli including thermal, electrical, chemical, and mechanical. In order to properly assess acute pain, it is imperative that the noxious stimulus is quantifiable, reproducible, and non-invasive. Furthermore, the stimulation must be delivered promptly and briefly enough to induce synchronous excitation of the nerve fibers [1]. For this reason, chemical stimulation is inadequate for studying acute pain. Similarly, different types of nociceptive stimuli are known to recruit distinct circuitry and induce pain with more-or-less specificity [1–3]. For instance, assays utilizing mechanical stimuli (e.g., von Frey) may activate innocuous afferents that could confound the observed pain response [2, 3]. Thermal stimulation, however, models a naturally-encountered stimulus, and its ability to induce pain with a relatively high degree of specificity contributes to its wide utilization throughout pain research [1].

The tail immersion assay is an assessment of thermal pain that is often used to determine the analgesic properties of drugs. The assay measures pain sensitivity by immersing a rat’s tail in hot water and recording the tail withdrawal latency, or the time it takes before a tail flick (i.e., pain response) is observed. Although there are now devices that automate data collection for numerous methods of pain measurement [4], most studies that use the tail immersion assay continue to rely on manual recording of latency via stopwatch. In addition, the temperature of the water is typically not monitored to control against potentially confounding fluctuations across measurements. By inviting increased technical variation, the use of manual methods likely contribute to some of the inconsistent findings among previous pain studies [5, 6]. Thus, the development of a more reliable method for this simple assay is warranted.

The current study describes our open-source device, *TailTimer*, which collects tail withdrawal latency and water temperature measurements automatically. The device uses a single-board computer (Raspberry Pi 3, RP3), which offers an affordable alternative to desktop computers. In addition, RP3 can be paired with a variety of devices and sensors to offer control over a wide range of environmental variables. Here, we added a digital temperature probe and a radiofrequency identification (RFID) system. Each injectable RFID chip contains a unique code that serves as the ID of a rat, which can be retrieved by a scanner connected to *TailTimer* via USB. This automated method by *TailTimer* abates the burden of data recording while, more importantly, ensuring precise measurement of tail withdrawal latencies and water temperature. Our device further enhances data collection standards by enabling the tail immersion assay to be conducted by a single technician, as opposed to the two that are typically needed for manual data collection (one must hold the rat while the other operates the stop watch). Thus, our *TailTimer* device offers an innovative solution for data collection in the tail immersion assay, thereby enhancing the reproducibility of tail withdrawal data.

## Materials and methods

### Study design

Here, we provide a detailed description of our *TailTimer* device so that it can be constructed by others. We then provide data to illustrate the sensitivity of the device by testing rats under different water temperatures and mixing speeds. Lastly, we evaluate the reliability and validity of the device for detecting commonly-evaluated differences in thermal pain sensitivity, such as variation by strain and across different timepoints surrounding administration of oxycodone–a widely used oral analgesic. An experimental timeline is available as S1 Timeline.

### Construction of the device

**Fig 1:**
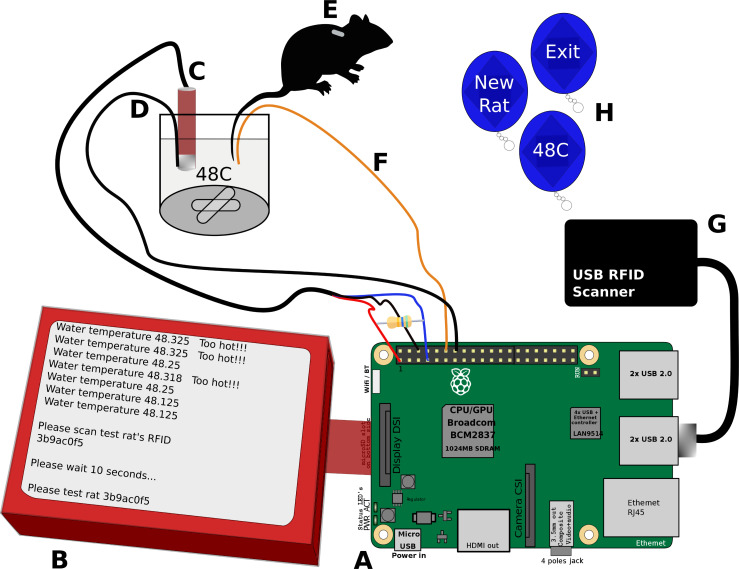
Components of the *TailTimer* device for use in the tail immersion assay. [**A**] The Raspberry Pi 3 computer. [**B**] The *TailTimer* software in operation. [**C**] Thermal probe to detect water temperature. [**D**] Electrical ground wire that remains immersed in the water with the thermal probe (C). [**E**] Scannable RFID implanted subcutaneously in the rat for identification. [**F**] Electrical latency wire to be dipped into and withdrawn from the water simultaneously with the rat’s tail to start and stop the timer, respectively. [**G**] USB RFID scanner. [**H**] Scannable RFID command fobs used to navigate the *TailTimer* program in place of a keyboard and mouse. The six necessary fobs are used to enter the user ID, set the target temperature, start a new rat, test the same rat again, delete the last latency, and exit the program.

*TailTimer* is composed of a Raspberry Pi 3 (Model B, RaspberryPi Foundation, UK) single-board computer (RP3), a 5-inch touch screen (DFR0550, DFRobot, ShangHai, China), a waterproof digital temperature sensor (DS18B20, Adafruit Industries, NY, USA), an RFID reader (EM4100, 125 kHz, HiTag, available at Amazon.com), and two electrical wires. All components are enclosed in a 3D-printed case. The connection of the components is illustrated in Fig 1 and the S1 Photo. The temperature sensor connects to the RP3 via a 1-wire serial interface to provide continuous temperature readings. The RFID reader connects to the RP3 via a USB port. It is used to identify individual rats by scanning the unique RFID chip embedded under their skin. The ground wire is connected to one of the ground pins of the general-purpose input/output (GPIO), and the latency wire is connected to GPIO 18 (pin 12). During operation, the ground wire and thermal probe remain immersed together in the hot water at a depth of approximately 4 cm. The latency wire is dipped into the hot saltwater solution at the same time as a rat’s tail and is taken out of the hot water, together with the tail, when a pain response is observed (i.e., the tail starts to “flick” in response to heat). *TailTimer* automatically records the precise times at which the latency wire contacts and is withdrawn from the water to calculate the tail withdrawal latency.

#### Hot water

A standard hot plate (Thermolyne Cimarec 1, Model SP46615) was used to heat a 1 L beaker containing 900 ml tap water. The target water temperature was 48°C for most experiments (see below for details of each experiment). To ensure homogeneity of the temperature, a 2 cm magnetic stir bar was used to mix the water at a consistent, low speed (setting 1 on the hot plate). Approximately 4 g of NaCl (0.44%) was added to increase conductivity of the water, as required for automated recording of the open/close state between the ground and latency wires.

### Software features, user interface, and testing procedure

*TailTimer* has a computer program written in the Python language. It collects information from the temperature probe and the RFID reader and logs the time during which the circuit between the latency wire and the ground wire is in the close state (i.e., tail withdrawal latency). A text-based user interface is automatically started when the device is powered on. It first prompts the user to input a username, set the target water temperature and mixing speed, and test the connection of the wires. Measures of tail withdrawal latency are then initiated by entering the ID of the rat. This can be achieved by scanning the RFID embedded under the skin of the rat using the USB RFID reader or by entering the ID via a keyboard. The interface then prompts the user to test the rat. During testing, the test rat is able to perch, unrestrained on the technician’s left arm. The user’s right hand is used to clinch the latency wire abreast the test rat’s tail for simultaneous immersion and subsequent withdrawal. The latency wire and tail, clinched together, remain immersed in the hot water at a depth of approximately 4 cm until a tail flick (i.e., pain response) is observed. The pain response is followed by prompt, simultaneous removal of the latency wire and tail from the water; this stops the circuit-based timer, and the number of seconds it took for the rat to elicit a pain response (i.e., the tail withdrawal latency) is automatically recorded by and displayed upon RP3. See S1 Video for demonstration. Each rat is tested at least twice. Additional testing is conducted when the difference between a rat’s latencies exceeds 1 s. Tails are removed from the water if the rat fails to elicit a response after 10 s. The software enforces an inter-trial interval of 10 s in between tests [7] and only allows testing to occur when the water is within ± 0.25°C of the target temperature. The device produces continuous water temperature updates and displays warnings when the temperature falls out of the target range. All relevant data are automatically recorded by the program. The user can operate the program using a keyboard; however, the RFID system offers a more convenient alternative by encoding the limited number of answers to the user interface questions using RFID fobs, where each fob represents one unique answer. A total of six fobs (one each for entering the user ID, setting the target temperature, starting a new rat, testing the same rat again, deleting the last latency, and exiting the program) are needed for all operations. Data gathered from the device are transferred using a standard USB storage drive.

#### Software and code accessibility

The software and design of the 3D enclosure described in the paper are freely available from the GitHub repository at https://github.com/chen42/tailTimer under an MIT license and registered with SciCrunch as RRID:SCR_021277. They are additionally available as S1 Code.

### Animals

The study was conducted on three-month-old, naïve males, comprised of three strains: Sprague Dawley (SD, *n* = 8), Wistar Kyoto less-immobile (WLI, *n* = 7), and Wistar Kyoto more-immobile (WMI, *n* = 6). A separate set of adult SD males (*n* = 5) were used to compare latencies measured by *TailTimer* against those collected manually via stopwatch. Breeders of the WLI and WMI strains were obtained from Dr. Redei, and the SD rats were purchased from Charles River Laboratories. The rats were housed in cages of three–four and maintained at The University of Tennessee Health Science Center in a temperature-controlled room on a 12 h dark/light cycle (lights on at 9:00 pm). Food and water were provided *ad libitum*. No enrichment items were provided. Testing was conducted across five consecutive days and initiated at 11:30 am each morning. The rats were tested in the same order throughout testing. In order to avoid possible confounding effects caused by signals transmitted among rats, each rat was placed in a holding cage after receiving a gavage and after completing testing at each timepoint. No animals were excluded. Following the conclusion of testing, the rats were used in subsequent experiments and eventually sacrificed under isoflurane anesthesia via cervical dislocation. All procedures were approved by the Animal Care and Use Committee of The University of Tennessee Health Science Center and were conducted in accordance with the NIH Guidelines concerning the Care and Use of Laboratory Animals.

#### RFID implantation

One RFID chip was implanted subcutaneously into each rat at the time of weaning. The RFIDs were soaked in 70% EtOH overnight and subsequently air dried. Each RFID was then loaded into a 16G needle, attached to an injector syringe. After cleaning the rat’s skin at the nape with 70% EtOH, the RFID was injected subcutaneously using the injector. Anesthesia was not needed for this procedure.

### Measuring tail withdrawal latency under different conditions

Baseline pain thresholds were determined by averaging each rat’s latency measures collected over two consecutive days. On day three of testing, *TailTimer* was used to assess the pain thresholds of the SD males (*n* = 8) at various water temperatures and mixing speeds. We performed the tail immersion assay on each rat using three different water stirring settings: low speed (setting 1), high speed (setting 7), and still (setting turned off) at a temperature of 48 ± 0.25°C. We also tested these rats at adjusted target temperatures of 47, 49, and 50°C when the water was mixing at a constant low speed (setting 1). During testing, the animals were held without restraint. All tail immersion assessments were performed by the same female investigator.

### Calibration of *TailTimer* with the manual stopwatch method

We then proceeded to compare tail withdrawal latencies measured automatically by *TailTimer* versus manually via stopwatch. For this experimentation, we used a separate set of adult SD males (*n* = 5). For each rat, latency was measured three times using *TailTimer* while a separate technician simultaneously recorded each latency using a standard stopwatch.

### Oxycodone administration

On the fifth and final day of testing, we administered oxycodone (3 mg/kg) to the SD males (*n* = 8) via oral gavage. One day prior (day four), the rats received oral gavages of distilled water as the control. All gavages were administered by one male technician, and the rats received their gavages of water and oxycodone in the same order. All rats were subsequently tested via the tail immersion assay using *TailTimer* at 15 min, 1 h, and 4 h post-gavage. The rats were placed in a holding cage after each gavage and after being tested at each timepoint.

### Statistical analyses

All analyses were conducted on a laptop computer running macOS Catalina 10.15.7 using the R-statistical software (version 1.3.1093). Data were presented as means ± SEM. Differences were considered significant at *p* < 0.05. Two separate ANOVAs with repeated measures were performed to test for main effects of water temperature with four levels (47, 48, 49, and 50°C) and water mixing speed with three levels (high, low, and still) on tail withdrawal latency. The relationship between water temperature and tail withdrawal latency was further assessed via linear regression analysis using the *lm* function. A paired t-test was performed to assess latencies measured by *TailTimer* against those measured via stopwatch. Another repeated-measures ANOVA was conducted to evaluate the relationship between oral oxycodone (or the distilled water control) on tail withdrawal latency, and Tukey HSD *post hoc* tests were performed to contrast the effects at 15 min, 1 h, and 4 h post-gavage. To test for possible differences in baseline latency by strain, we performed a between-groups ANOVA with three levels (SD, WLI, and WMI) followed by Tukey HSD *post hoc* for comparison.

## Results

Here we report the development of *TailTimer*, the first open-source device to offer automated quantification of tail withdrawal latency and monitoring of water temperature for use in the rodent tail immersion assay. We designed *TailTimer* to quantify tail withdrawal latency automatically by measuring the close time of a circuit between a ground wire that remains immersed in a conductive salt solution and a latency wire that is first dipped into and then withdrawn from the solution together with a tail when the tail starts to flick in response to heat.

### Water temperature influences tail withdrawal latency

**Fig 2:**
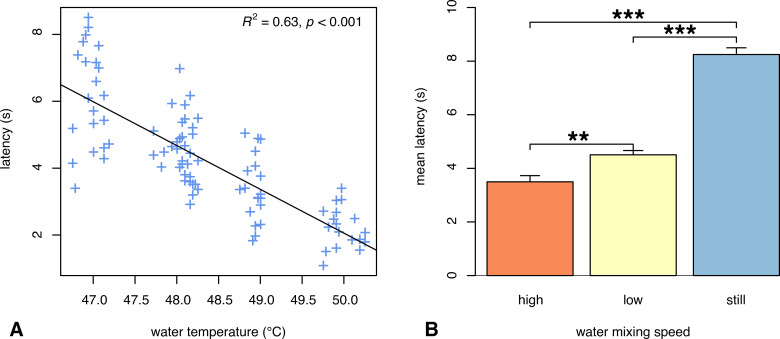
Latency varies across water conditions. [**A**] Tail withdrawal latency measured at different water temperatures. Each data point (+) represents one of the 2–3 latency measures obtained for each individual rat at each temperature tested. As water temperature is decreased, latency is lengthened. Linear regression indicated that temperature explains 63% of the variance in latency when tested between 47–50°C with the water mixing at a constant, low speed. [**B**] Tail withdrawal latency measured at a constant temperature (48°C ± 0.25) and different water mixing speeds. Lengthening of latency occurs as the water mixing speed is decreased with the longest latencies occurring when the water is still (i.e., not being mixed by the stir bar). Data are expressed as mean ± SEM; **p = 0.001, ***p < 0.001.

Baseline pain thresholds were determined by averaging each rat’s latency measures collected over two consecutive days. Results of a repeated-measures ANOVA showed no significant difference in latency between days for each rat, indicating stable baseline measures. *TailTimer* was then used to assess pain thresholds at four different water temperatures (47, 48, 49, and 50°C). Results of a repeated-measures ANOVA revealed a significant main effect of water temperature on tail withdrawal latency, *F*_(3,21)_ = 43.55, *p* < 0.001, in which latencies were significantly longer at lower water temperatures and shorter at higher temperatures. Tukey HSD *post hoc* analysis indicated significant differences in mean latency among all temperature conditions. Interestingly, an especially great mean difference of 1.63 s was observed between latencies measured at 47 (6.14 ± 0.32 s) versus 48°C (4.5 ± 0.16 s), *p* < 0.0001. In contrast, the mean differences observed between 48–49 and 49–50°C were 1.15 s (*p* = 0.001) and 1.14 s (*p* = 0.007), respectively. As illustrated in Fig 2A, results of the linear regression analysis also revealed a significant relationship between water temperature and latency, *F*_(1,93)_ = 158.3, *p* < 0.001, with an *R*^*2*^ of 0.63. The strong linear relationship indicated that the withdrawal latency measured by *TailTimer* is highly accurate.

### Water mixing speed influences tail withdrawal latency

During prior experimentation, we observed an effect of water mixing speed on tail withdrawal latency. Therefore, we systematically measured tail withdrawal latency on the eight SD males at 48°C under three different water mixing speeds. A repeated-measures ANOVA yielded a significant main effect of water mixing speed on tail withdrawal latency, *F*_(2,14)_ = 113.68, *p* < 0.001. *Post hoc* analysis (Fig 2B) revealed significantly shorter latencies at the high spin setting (3.5 ± 0.23 s) relative to the low (4.5 ± 0.16 s) and no spin (8.25 ± 0.25 s) conditions. Similarly, latencies were significantly longer when the water was still versus mixing at a low speed (*p* < 0.001). Together, these data elucidate the importance of maintaining consistent water mixing speed across experiments.

### Manual validation of latencies measured by *TailTimer*

A paired t-test revealed no significant difference between latencies measured by *TailTimer* (6.65 ± 0.39 s) versus those collected via stopwatch (6.48 ± 0.4 s), *t*(4) = 1.29, *p* = 0.27. These results demonstrate consistency among data collected automatically by *TailTimer* with that collected manually using the traditional stopwatch method.

### *TailTimer* detects oxycodone-induced changes in pain sensitivity

**Fig 3:**
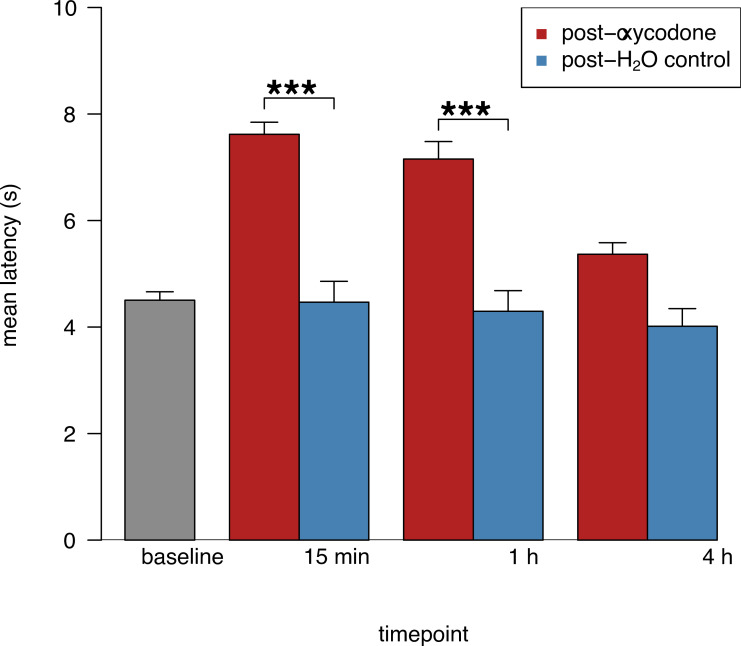
*TailTimer* detects acute changes in tail withdrawal latency following oral oxycodone exposure. Pain thresholds of SD males (*n* = 8) were evaluated at 15 min, 1 h, and 4 h post-gavage of 3 mg/kg oxycodone or distilled water at a controlled water temperature of 48°C ± 0.25 and a fixed, low water mixing speed (setting 1). Latencies were significantly longer at 15 min and 1 h, but not 4 h, post-gavage of oxycodone versus distilled water. Following gavage of the distilled water control, latencies were not significantly different from baseline at any time point. Mean latencies reflect the average of the (two–four) measurements per individual rat averaged across timepoints. Data are expressed as mean ± SEM; ***p < 0.001.

We next used *TailTimer* to probe for changes in thermal pain sensitivity following acute administration of oxycodone. We measured tail withdrawal latency in the SD males at three timepoints (15 min, 1 h, and 4 h) following oral gavage of oxycodone or a distilled water control. Results of a repeated-measures ANOVA revealed significant main effects of drug condition, *F*_(1,5)_ = 59.54, *p* < 0.001, and timepoint, *F*_(3,18)_ = 11.65, *p* < 0.001, on tail withdrawal latency. More importantly, there was a significant interaction between timepoint and drug condition, *F*_(2,14)_ = 18.61, *p* < 0.001. *Post hoc* analysis (Fig 3) indicated significantly longer latencies following gavage of oxycodone versus distilled water at 15 min (7.62 ± 0.39 s) or 1 h (7.15 ± 0.39 s), but not 4 h (5.37 ± 0.33 s). No significant difference was observed between latencies obtained 15 min versus 1 h post-oxycodone. Importantly, no significant effects were observed following gavage of the distilled water control at any of the three timepoints. Together, these findings valorize *TailTimer* in its ability to detect anxiolytic-induced changes in thermal pain sensitivity across time.

### Biological differences in pain sensitivity can be detected by *TailTimer*

**Fig 4:**
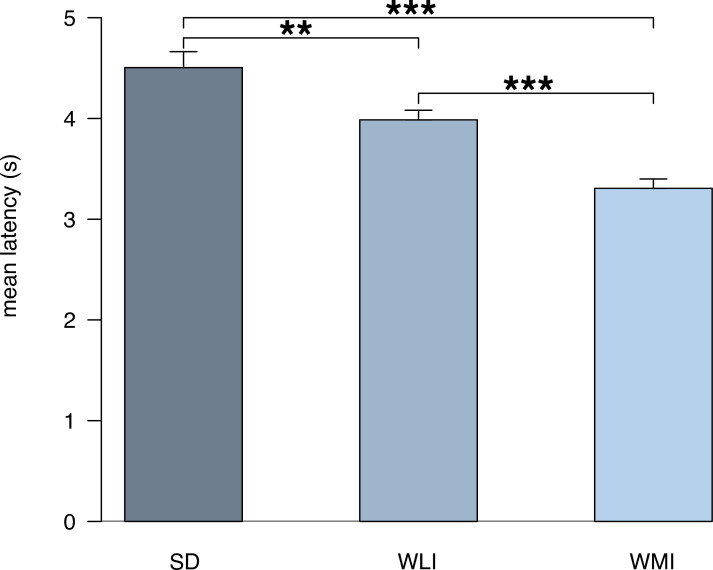
*TailTimer* detects strain differences in baseline pain thresholds. The tail immersion assay was performed to determine baseline tail withdrawal latencies for males of the outbred SD strain (*n* = 8) and of the WLI (*n* = 7) and WMI (*n* = 6) inbred strains. Baseline measurements were collected for each rat on two separate, consecutive days. Mean latencies reflect the average of the (four–eight) tests per individual rat, averaged across strain. Data are expressed as mean ± SEM; **p = 0.006, ***p < 0.001.

We proceeded to use *TailTimer* to detect strain differences in thermal pain sensitivity by measuring tail withdrawal latency in males of outbred (SD) and inbred (WLI and WMI) strains. Results of a one-way ANOVA revealed a significant main effect of strain, *F*_(2,99)_ = 19.76, *p* < 0.001. Interestingly, SD rats exhibited significantly longer latencies (4.5 ± 0.16 s) compared to the WLI (3.99 ± 0.1 s) and WMI strains (3.31 ± 0.09 s). *TailTimer* also detected a significant difference between the two, isogenic inbred strains in which longer latencies were observed in the WLI versus WMI rats (*p* < 0.001). These results are illustrated in Fig 4.

## Discussion

The purpose of the current study was to develop an automated device for measuring pain sensitivity using the tail immersion assay. *TailTimer’s* hardware design and software program are both open source, and the device can be assembled using inexpensive parts. *TailTimer* is simple to operate and requires only one person to handle the entire data collection process. Importantly, the data collected automatically by *TailTimer* are consistent with those collected manually by two trained technicians. Another major advantage is that, for each tail withdrawal latency data point, *TailTimer* automatically captures multiple variables as meta data, including animal ID, date, time, name of technician, water temperature at the time of each measurement, and water mixing speed. These meta data enhance the value of the primary data and improve the scientific rigor of the assay.

The results of our experimentation using an assortment of target temperatures (47, 48, 49, and 50°C) demonstrate the effect of temperature on tail withdrawal latency. As shown in Fig 2A, we found a significant negative relationship between increasing water temperature and latency, whereby a 1-degree difference in temperature significantly affects latency. Because tail withdrawal latency is determined by water temperature, we designed the *TailTimer* software to only allow tail withdrawal latency to be measured within a limited range of ± 0.25°C, thereby minimizing the technical variance.

Although it is not commonly reported, water mixing speed is likely another factor contributing to discrepancies among previous literature. Higher water mixing speeds are concomitant with increased rates of heat exchange, which can thereby accelerate the speed at which thermal pain is induced. To test this effect, we performed the tail immersion assay on eight SD males at different water mixing speeds (high, low, and still) at 48°C ± 0.25. As shown in Fig 2B, we observed significantly shorter latencies at faster water mixing speeds with the longest latencies revealed when the water was not being mixed (i.e., still). These findings elucidate the importance of standardizing water mixing speed throughout subsequent experimentation using the tail immersion assay.

Moreover, it is worth noting that our testing procedures do not include methods of restraining rats that have been utilized across many extant tail immersion studies. The use of restraining methods during testing has been shown to induce additional stress and consequently affect tail withdrawal latency [8, 9]. For this reason, we chose to hold the rats without restraint throughout testing.

The tail immersion assay is commonly used to evaluate the analgesic properties of drugs. Therefore, we proceeded by conducting an experimental analgesic drug study to evaluate *TailTimer* in its ability to detect oxycodone-induced changes in pain sensitivity. We used *TailTimer* to test the SD rats’ tail withdrawal latencies at 15 min, 1 h, and 4 h post-gavage of the distilled water control (day four) and oxycodone (day five). Compared to baseline, latencies were significantly longer following oxycodone administration when measured after 15 min and 1 h but not after 4 h. These results (Fig 3) would be expected following acute administration, as the analgesic effects of oral oxycodone have been shown to peak in rodents after 15 min, persist for 1–2 h, and extinguish by 4 h [10]. Moreover, latency did not significantly differ from baseline at any time point following oral gavage of the distilled water, indicating little to no influence of potential stress associated with gavage. These findings demonstrate the ability of *TailTimer* to detect opioid-induced changes in thermal pain sensitivity across time. Using *TailTimer*, we also found significant strain differences in baseline tail withdrawal latency. As shown in Fig 4, significant differences were observed between each strain in which SD and WMI rats exhibited the longest and shortest latencies, respectively. These results evidence *TailTimer’s* ability to detect genetically determined differences in thermal pain sensitivity.

One innovative aspect of *TailTimer* is the use of RFID as the primary user data input device. Rodent behavioral tests are usually conducted in tight spaces where gloves are mandatory. This unique work environment poses challenges for using a keyboard/mouse combination or touch screens as primary data input devices. We therefore adapted the RFID system, primarily used to record the identity of the animals, for use as the primary user interface which can be operated by scanning unique RFID fobs. This implementation provides a convenient solution for entering predetermined information (e.g., user ID, target temperature, water mixing speed) and navigating the program (e.g., progressing to the next step, repeating the measure, exiting the program). The RFID reader we use has a USB interface that is easy to program. Although a keyboard can still be used to operate *TailTimer*, we almost always employ the RFID system because of its convenience.

*TailTimer* is one of the many laboratory devices that uses the Raspberry Pi single-board computer. These computers encompass computing power that rivals desktop computers of the last decade at a small fraction of their predecessors’ size and cost. A large part of the appeal of these computers is the vast array of external devices (e.g., sensors or motors) that can be connected and controlled through the GPIO ports. Devices for operant conditioning [11, 12], conditioned place preference [13], head-fixed mesoscale cortical imaging [14], and even virtual reality [15] have been reported. Further, a wide range of environmental factors (e.g., humidity, barometric pressure, and light) can be monitored using Raspberry Pi [12]. Although some technical know-how is needed for making these devices, detailed instructions are generally available. Most of these devices are open source and can therefore be modified to fit new requirements. For example, it is feasible to use *TailTimer* with some modification to measure the rectal temperature of rodents.

Notably, *TailTimer* is not without limitations. A technician still needs to observe the movement of the tail carefully and decide when to withdraw the tail and wire from the solution. To ensure consistency, a single technician should be designated to administer all assessments. In addition, some technical know-how are needed to 3D print the case, assemble the device, and install the software. It is also necessary to cross validate the accuracy of the digital temperature probe. This is even more critical when multiple setups are used in the same lab. Moreover, while a standard hot plate has been utilized as the go-to heating source across decades of tail immersion studies, subsequent research may elect to employ a more advanced alternative that can provide increased convenience and further abate the degree of variability in water temperature across tests. Since the conclusion of testing for this study, we have adopted a digital stirring hot plate (Thermo Scientific RT2 Advanced Hotplate Stirrer, Catalog #88880006) that offers increased temperature stability at ± 0.1°C and reduces the amount of time required for the water to reach the target temperature. Although not strictly a limitation of *TailTimer*, choosing an optimal water temperature is also critical for obtaining valid data. While we have used 48°C in our tests, studies should consider how the optimal temperature may vary based on the goal of the study.

## Conclusions

In summary, we report an open-source, simple, and inexpensive device for measuring the tail withdrawal latency of rodents using the tail immersion assay. This device automatically records latencies, water temperature, and the identifications of both animals and technicians. It also limits the tests to be conducted only when the water is within ± 0.25°C of the target temperature. We anticipate the increased ease of operation and reproducibility of tail immersion assay data, provided by *TailTimer*, to augment its utility in addiction and nociception research.

## Supporting information

S1 Photo *TailTimer* device components. (TIFF)

S1 Video *TailTimer* in operation. (MOV)

S1 Timeline Experimental timeline. Baseline latency measures were collected via *TailTimer* across the first two consecutive days. On the following day, *TailTimer* was used to measure tail withdrawal latency in the SD males under four different water temperatures (47, 48, 49, and 50°C) while the water mixed at a low, fixed rate. Next, latencies were measured under high, low, and still water mixing speed conditions while the temperature was held constant at ± 0.25°C. On days 4 and 5, tail withdrawal latencies were measured via *TailTimer* in the SD males at 15 min, 1 h, and 4 h following oral gavage of distilled water or oxycodone (3 mg/kg), respectively.
(TIF)

S1 Codes *TailTimer* software code and 3D case design. Four files containing the codes for the *TailTimer* program (tailwithdrawal.py), running the program on system start-up and copying the data to USB drive after exiting the program (run_tailtimer.sh), programming commands into RFID fobs for navigation (config.py), and the design of the 3D-printed case (tailtimer_case.scad).
(ZIP)

S1 Script Main R script from statistical analyses. (R)

S2 Script R script from calibration analysis. (R)

S1 Data Main tail withdrawal latency data file. All data collected from our sample analgesic drug study and baseline latency comparison across strains.
(CSV)

S2 Data *TailTimer* versus stopwatch calibration data set. (CSV)
