# Supplementary figures and images for: TailTimer: A device for automating data collection in the rodent tail immersion assay
Source: PLoS One. 2021 Aug 19;16(8):e0256264. doi: 10.1371/journal.pone.0256264 (PMC8375991; doi:10.1371/journal.pone.0256264)

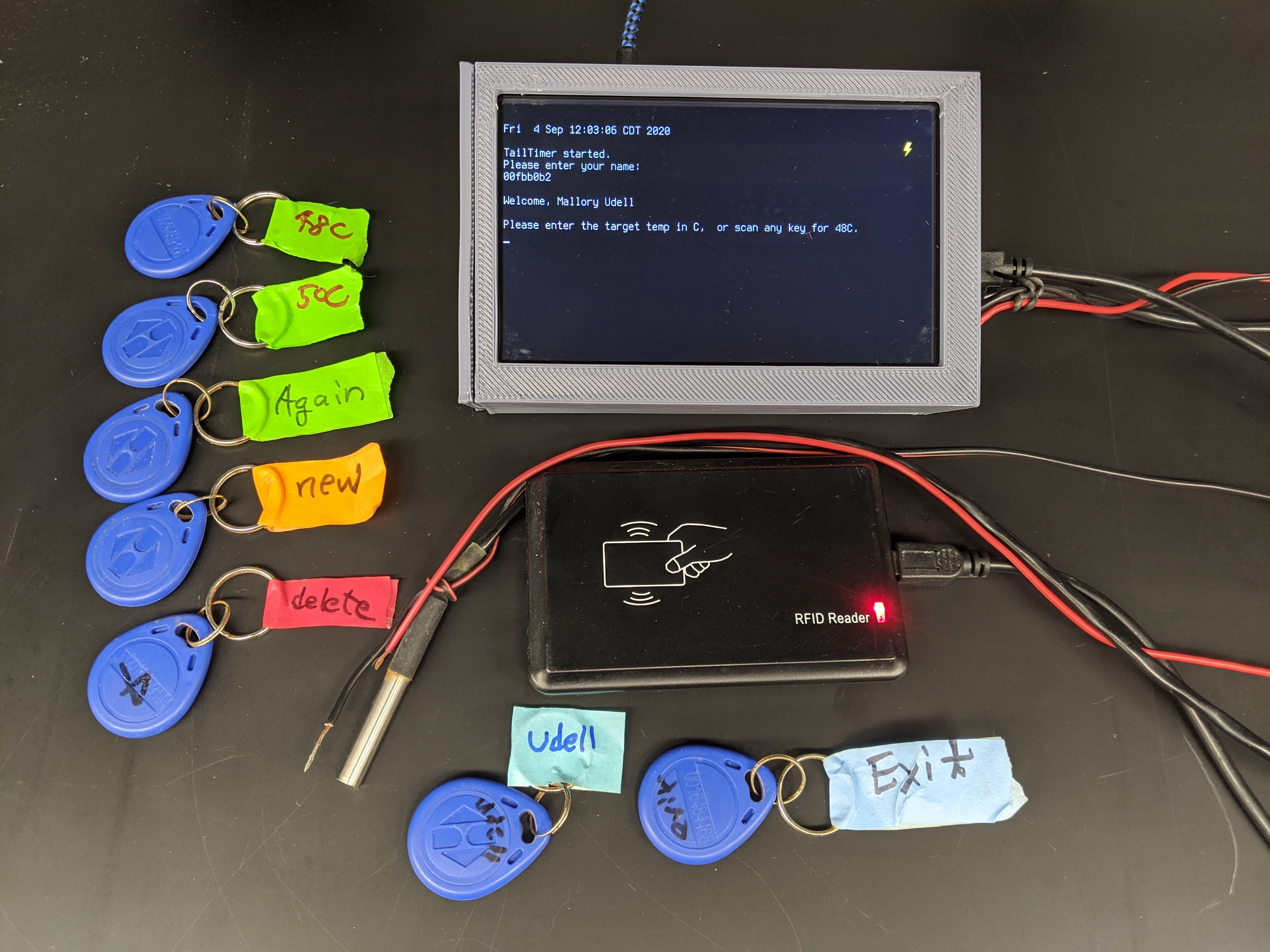

Supplement: S1 Photo — (TIFF) [file pone.0256264.s001.tiff]

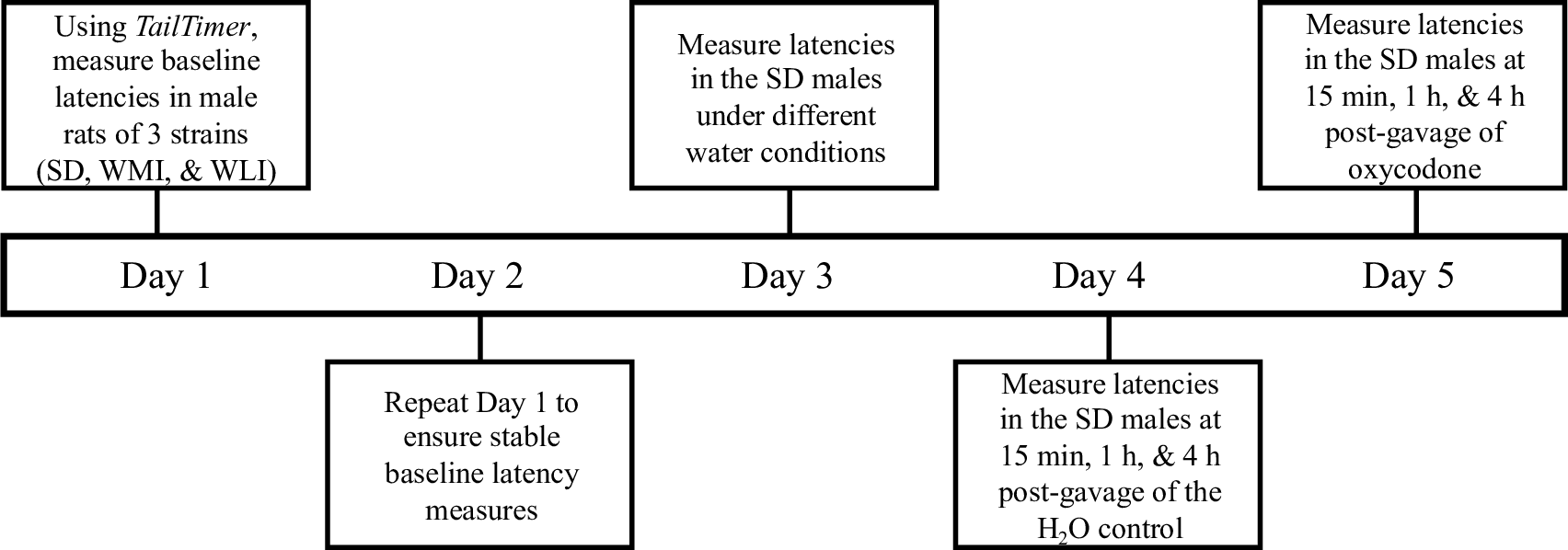

Supplement: S1 Timeline — Baseline latency measures were collected via TailTimer across the first two consecutive days. On the following day, TailTimer was used to measure tail withdrawal latency in the SD males under four different water temperatures (47, 48, 49, and 50°C) while the water mixed at a low, fixed rate. Next, latencies were measured under high, low, and still water mixing speed conditions while the temperature was held constant at ± 0.25°C. On days 4 and 5, tail withdrawal latencies were measured via TailTimer in the SD males at 15 min, 1 h, and 4 h following oral gavage of distilled water or oxycodone (3 mg/kg), respectively. (TIF) [file pone.0256264.s003.tif]
